# The Development and Application of a Dot-ELISA Assay for Diagnosis of Southern Rice Black-Streaked Dwarf Disease in the Field

**DOI:** 10.3390/v4010167

**Published:** 2012-01-23

**Authors:** Zhenchao Wang, Dandan Yu, Xiangyang Li, Mengjiao Zeng, Zhuo Chen, Liang Bi, Jiaju Liu, Linhong Jin, Deyu Hu, Song Yang, Baoan Song

**Affiliations:** 1 State Key Laboratory Breeding Base of Green Pesticide and Agricultural Bioengineering/ Key Laboratory of Green Pesticide and Agricultural Bioengineering, Ministry of Education, Guizhou University, Guiyang, 550025, P. R. China; Email: gs.zcwang@mail.gzu.edu.cn (Z.C.W.); dandanyu0813@hotmail.com (D.D.Y.); gs.lixy09@mail.gzu.edu.cn (X.Y.L.); fcc.zchen@gzu.edu.cn (Z.C.); gs.lbi09@mail.gzu.edu.cn (L.B.); liujiaju1987@126.com (J.J.L.); fcc.jinlh@gzu.edu.cn (L.H.J.); fcc.dyhu@gzu.edu.cn (D.Y.H.); 2 School of Chemistry and Chemical Engineering, South China University of Technology, Guangzhou, 510640, P. R. China; Email: 1094185612@qq.com (M.Z.)

**Keywords:** SRBSDV, detection, dot-ELISA, One Step RT-PCR, county laboratories

## Abstract

Outbreaks of the southern rice black-streaked dwarf virus (SRBSDV) have caused significant crop losses in southern China in recent years, especially in 2010. There are no effective, quick and practicable methods for the diagnosis of rice dwarf disease that can be used in the field. Traditional reverse transcription-polymerase chain reaction (RT-PCR) methodology is accurate but requires expensive reagents and instruments, as well as complex procedures that limit its applicability for field tests. To develop a sensitive and reliable assay for routine laboratory diagnosis, a rapid dot enzyme-linked immunosorbent assay (dot-ELISA) method was developed for testing rice plants infected by SRBSDV. Based on anti-SRBSDV rabbit antiserum, this new dot-ELISA was highly reliable, sensitive and specific toward SRBSDV. The accuracy of two blotting media, polyvinylidene fluoride membrane (PVDF membrane) and nitrocellulose filter membrane (NC membrane), was compared. In order to facilitate the on-site diagnosis, three county laboratories were established in Shidian (Yunnan province), Jianghua (Hunan Province) and Libo (Guizhou province). Suspected rice cases from Shidian, Yuanjiang and Malipo in Yunnan province were tested and some determined to be positive for SRBSDV by the dot-ELISA and confirmed by the One Step RT-PCR method. To date, hundreds of suspected rice samples collected from 61 districts in southwestern China have been tested, among which 55 districts were found to have rice crops infected by SRBSDV. Furthermore, the test results in the county laboratories showed that Libo, Dehong (suspected samples were sent to Shidian) and Jianghua were experiencing a current SRBSDV outbreak.

## 1. Introduction

Southern rice black-streaked dwarf virus (SRBSDV) is currently one of the most damaging rice crop diseases in China. The SRBSDV is a novel member of Fijivirus (family Reoviridae) [1,2,3], first observed in Guangdong province in 2001 and identified in 2008. Wang reported the complete SRBSDV nucleotide sequences of isolates from Hainan and Guangdong provinces of China, and confirmed that it is a novel Fijivirus species [3]. Yin obtained an isolate of SRBSDV from naturally infected maize plants from Jining, Shandong province [4]. At present, SRBSDV mainly distributes in Southern Asia, including Vietnam [5,6], Japan and China. According to incomplete statistics, SRBSDV has caused extensive crop damage in Jiangxi, Hunan, Guangdong, Fujian, Hainan, Guizhou, and Yunnan since 2009 in China. Mid-late rice crops are affected more seriously than the early season crops in most districts [7,8,9,10]. Approximately 5 million acres of rice had been infected by this virus in China and 100,000 acres of rice farm reported crop failure in 2009 [11]. In 2010, nearly 3 million acres of rice were infected by SRBSDV, suggesting a rapid spread and major losses in the coming years [12]. 

Symptoms of SRBSDV infection vary depending on the crop age when infected. Characteristic symptoms include dark-green and wrinkled leaves, incomplete tassel, tumor-like protrusions ending in small enations, tiller formation on the upper parts, and up-growing rootlets [7,13,14]. Among these, the white tumor-like growths and ectopic tiller are the typical symptoms. The disease mainly injures gramineous plants such as rice, corn, wheat and grass weeds [15]. The SRBSDV is transmitted by the white backed plant hopper (WBPH) [1], and the main mode of transmission is the feeding process rather than spawning [16]. 

The current laboratory diagnosis of SRBSDV disease employs RT-PCR to detect viral RNA [17,18] in plant samples, which requires a trained laboratory technician. This process would be expensive, inconvenient and prone to contamination under field conditions [19,20,21]. The visible manifestations of SRBSDV infection are delayed, and it is difficult to prevent and control spread once symptoms are visible. Thus, an early detection is critical for disease control [22].

In the past several decades, immunoassays have become routine for detecting plant pathogens, both viruses and bacteria [23]. The dot-enzyme-linked immunosorbent assay (dot-ELISA) is a solid-phase immunoassay that can be used to detect either antigen or antibody [24,25,26]. Dot-ELISA is a simple, rapid and scalable procedure for screening a large number of samples at one time [27]. Optimization and standardization of reagents and test procedures for serodiagnosis of viral diseases are vital for assay validity, reproducibility and quality control [28]. In the present study, three polyclonal S10 antibodies were obtained and measured by indirect enzyme-linked immunosorbent assay and western blot analysis. Thus, a dot-ELISA approach was developed for the identification of novel serologically reactive SRBSDV antigens derived from infected rice plants [29]. The main purpose of the present study was to use the developing dot-ELISA technology to diagnose suspected SRBSDV infection in rice plants picked from Southwest China. To the authors’ knowledge, this is the first study to diagnose SRBSDV disease by using dot-ELISA method in field.

## 2. Results and Discussion

### 2.1. Optimization of dot-ELISA method

**Figure 1:**
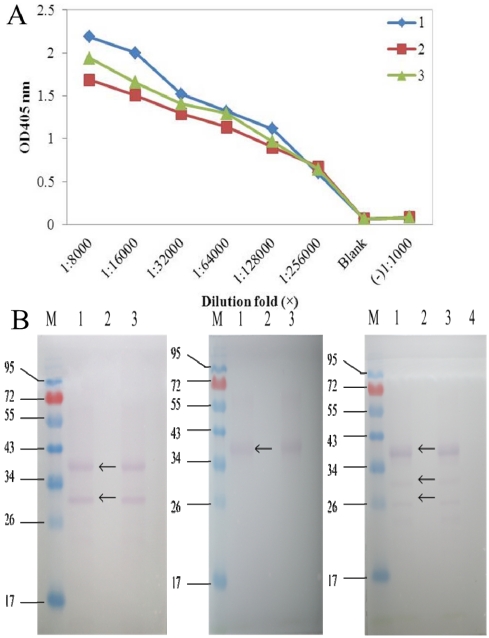
**(A)** Titers of the three SRBSDV S10 polyclonal antibodies. 1-3 represent antibodies 1, 2, 3 respectively. **(B)** Specificity analysis of the three SRBSDV S10 polyclonal antibodies 1, 2, 3 (from left to right).

**Figure 2:**
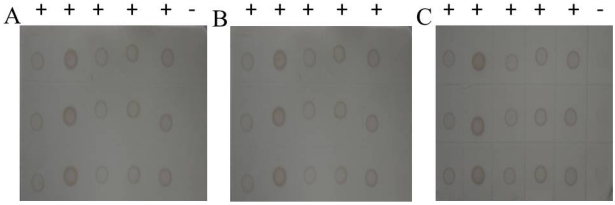
**(A)** 1:1000-fold antiserum dilution and 60 min second blocking time. **(B)** 1:1500-fold antiserum dilution and 60 min second blocking time. **(C)** 1:1500-fold antiserum dilution and 30 min second blocking time.

The titer results of S10 polyclonal antibodies evaluated via indirect ELISA showed that both S10 multi-antibody titers reached 1:500 000, which was an excellent ratio. S10 antibody 1 (diluted ratio of 1:8000) had the highest OD value (2.187), whereas antibody 2 had the lowest (1.684). Then specificity of S10 polyclonal antibodies was measured by Western blot examination (Figure 1), and we selected S10 polyclonal antibody 1 in our dot-ELISA assay. Preliminary experiments using viruliferous rice samples established the optimum dilutions of primary antibody needed for the dot-ELISA assay. Different dilutions of primary antibody in blocking buffer (1:1500 and 1:1000) and blocking time of primary incubation (60 min and 30 min) were tested (Figure 2). Satisfactory results were obtained with a 1:1500 dilution of the primary following 30 min of blocking time. The NC membrane was used to test rice samples infected by SRBSDV (except in the seeding stage because of the influence of plant pigment) and exhibited the same diagnostic results as PVDF membrane.

### 2.2. Test with dot-ELISA method in lab

**Figure 3:**
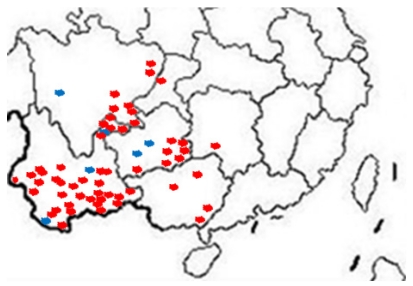
Distribution of SRBSDV infection in China (2011). Red indicates the samples infected with SRBSDV, the blue ones represent those uninfected.

**Figure 4:**
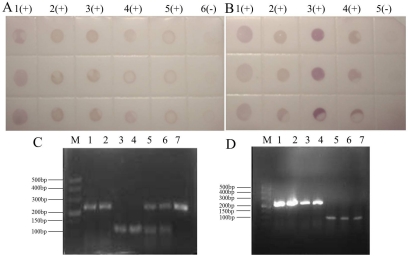
Positive controls were rice plants from Libo Guizhou province with confirmed SRBSDV infection. Negative controls were healthy rice plants grown in a greenhouse. **(A)** Dot-ELISA test (PVDF membrane) results of suspected rice plants from Shidian and Yuanjiang in Yunnan province. 1(+): Positive control. 2(+): Sample I from Shidian. 3(+): Sample II from Shidian. 4(+): Sample I from Yuanjiang. 5(+): Sample II from Yuanjiang. 6(-): Negative control.** (B) **Dot-ELISA test (PVDF membrane) results of suspected rice plants from Malipo Yunnan province. 1(+): Positive control. 2(+): Sample I. 3(+): Sample II. 4(+): Sample III. 5(-): Negative control. **(C) **One Step RT-PCR test results of suspected rice plants from Shidian and Yuanjiang. M: M, DL 500 bp DNA marker. 1: S10 gene amplification of rice sample I from Yuanjiang. 2: S10 gene amplification of rice sample IV from Shidian. 3: Reference gene amplification of rice sample I from Yuanjiang. 4: Reference gene amplification of rice sample IV from Shidian. 5: Multiple PCR of sample I from Yuanjiang. 6: Multiple PCR of sample IV from Shidian. 7: S10 gene amplification of positive sample. **(D) **One Step RT-PCR test results of suspected rice plants from Malipo. M: M, DL 500 bp DNA marker. 1: S10 gene amplification of rice sample I. 2: S10 gene amplification of rice sample II. 3: S10 gene amplification of rice sample III. 4: Positive control. 5: Reference gene amplification of rice sample I. 6: Reference gene amplification of rice sample II. 7: Reference gene amplification of rice sample III.

Our group tested hundreds of suspected SRBSDV rice samples from more than 60 districts in southwestern China from June to August 2011 (Figure 3), and randomly chosen results were confirmed by the One Step RT-PCR assay. In the One Step RT-PCR assay with primers S10F/S10R, the unique band of SRBSDV occurred at 242 bp (Figure 4).

**Figure 5:**
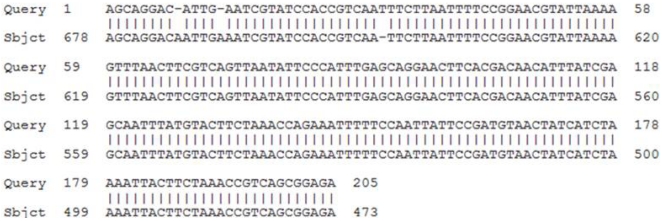
Comparative genomic sequence of SRBSDV between Shidian and Shaxian.

Furthermore, the amplified SRBSDV genomic sequences from Shidian were analyzed (Figure 5), and the homology of SRBSDV gene sequences between Shidian and Shaxian (retrieved from Genbank) in Fujian province was 99%. A purplish red dot illustrate that samples were infected with SRBSDV (Figure 4). Based on comparisons, the dot-ELISA method was as accurate as the One Step RT-PCR assay for detecting infection in rice samples from Shidian, Yuanjiang and Malipo counties.

**Figure 6:**
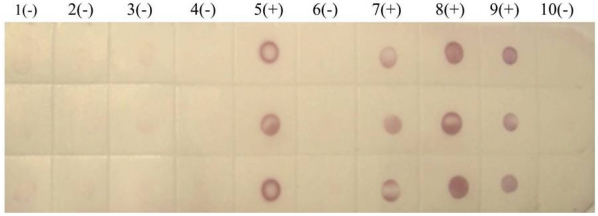
Dot-ELISA test (PVDF membrane) results of suspected rice plants from Yunnan province. 1(-): Sample I from Menghai. 2(-): Sample II from Menghai. 3(-): Sample III from Menghai. 4(-): Sample IV from Menghai. 5(+): Sample from Shidian. 6(-): Sample I from Lincang. 7(+): Sample II from Lincang. 8(+): Sample from Chuxiong. 9(+): Positive control. 10(-): Negative control.

We also tested rice samples by dot-ELISA using PVDF membranes. Again the purple-red spots indicated that the tested rice strains from Lincang (one strain) and Chuxiong (Figure 6) were infected with SRBSDV, while a sample from Menghai was not.

**Figure 7:**
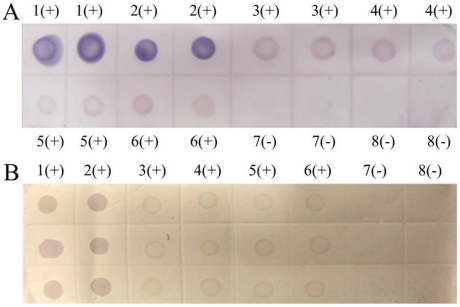
**(A)** Dot-ELISA test (PVDF membrane) results from Tianzhu Guizhou Province and Liangping Chongqing City. 1(+): Positive control. 2(+): Positive control. 3(+): Sample I from Tianzhu. 4(+): Sample II from Tianzhu. 5(+): Sample III from Tianzhu. 6(+): Sample from Liangping. 7(-): Negative control (stem). 8(-): Negative control (root). **(B) **Dot-ELISA test (NC membrane) results from Tianzhu and Liangping. 1(+): Positive control. 2(+): Positive control. 3(+): Sample I from Tianzhu. 4(+): Sample II from Tianzhu. 5(+): Sample III from Tianzhu. 6(+): Sample from Liangping. 7(-): Negative control (root). 8(-): Negative (stem).

The sensitivities of PVDF and NC membrane were compared by parallel testing of samples from Tianzhu in Guizhou Province and Liangping in Chongqing City. The detection results of dot-ELISA were both positive (Figure 7) but light purple spot indicated that the virus contents of the samples were lower than the positive control. Given that there was no difference in sensitivity between membranes, we selected the cheaper NC membrane for high throughput detection of SRBSDV infection.

**Figure 8:**
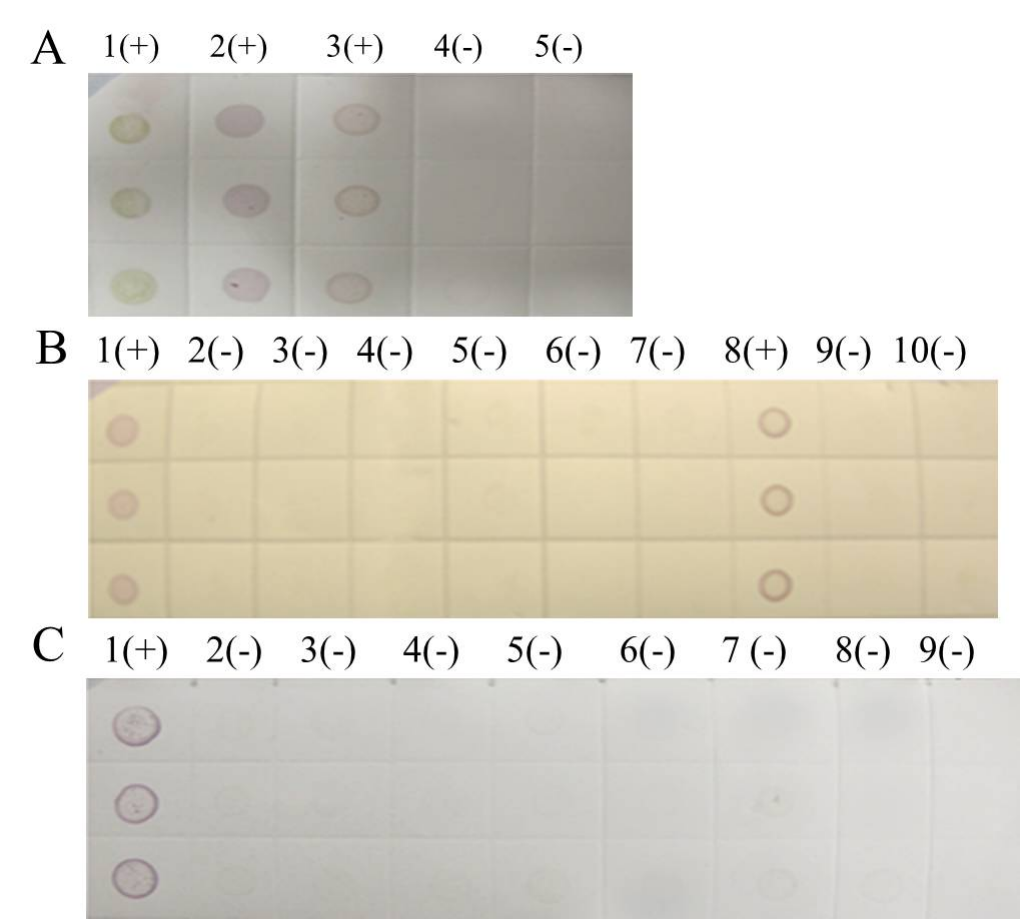
Dot-ELISA test (NC membrane) results of suspected rice plants. **(A)** Luodian of Guizhou Province. 1(+): Positive control. 2(+): Sample from Dongjia in Luodian. 3(+): Sample from Bianyang in Luodian. 4(-): Negative control of root. 5(-): Negative control of stem. **(B) **Jiajiang of Sichuan province and Dushan of Guizhou Province. 1(+): Positive control. 2(-) - 7(-): Stem of sample from Jiajiang. 8(+): Sample from Dushan. 9(-): Negative control of root. 10(-): Negative control of stem. **(C) **Kaiyang and Huishui of Guizhou Province. 1(+): Positive control. 2(-): Root of sample from Kaiyang. 3(-): Stem sample from Kaiyang. 4(-): Stem of sample I from Huishui. 5(-): Root of Sample I from Huishui. 6(-): Root of Sample II rom Huishui. 7(-): Stem of sample II from Huishui. 8(-): Negative control of stem. 9 (-): Negative control of root.

**Figure 9:**
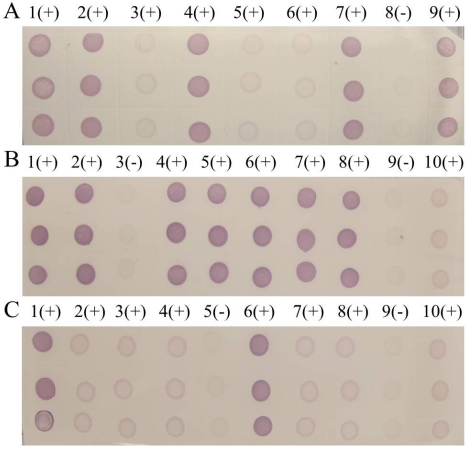
Dot-ELISA test (NC membrane) results of suspected rice plants from Yunnanand Sichuan provinces. **(A)** 1(+) – 7(+): Kaiyuan, Jinghong, Shizong, Yongshan, Mile, Pingbian and Mengzi respectively. 8(-): Negative control of stem. 9(+): Positive control. **(B)** 1(+): Positive control. 9(+): Negative control of stem. 2(+) – 10(+): Mengla, Ludian, Gejiu, Yiliang, Fengqing, Shuifu, Zhenxiong, Yanjin. **(C)** 1(+): Positive control. 9(+): Negative control of stem. 2(+) – 10(+): Maguan, Jiangcheng, Eshan, Kunming, Qiubei, Yuxi, Fushun, Hejiang.

The rice samples from 61 regions of Guizhou, Sichuan, and Yunnan province were tested in our laboratories, and 55 districts had at least one infected sample. Only selected results were discussed in this paper. The noticeable purple-positive spots of the samples from Luodian and Dushan in Guizhou province showed that these areas were experiencing an outbreak of SRBSDV disease (Figure 8). In addition, samples from Kaiyuan, Jinghong, Shizong, Yongshan, Mile, Pingbian, Mengzi, Mengla, Shiping, Gejiu, Jianshui, Yiliang, Xinping, Fengqing, Shuifu, Zhenxiong, Yanjin, Maguan and Guangnan in Yunnan province were also positive for SRBSDV disease (Figure 9). In contrast, there were no obvious spots on tests of samples from Huishui and Kaiyang in Guizhou province, Jiajiang in Sichuan province (Figure 8), or Ludian and Kunming in Yunnan province (Figure 9).

### 2.3. Diagnosis with dot-ELISA method in three county laboratories

During the same period, fast check-test technology for SRBSDV was applied in three county laboratories built by our group in Libo (Guizhou province), Shidian (Yunnan province) and Jianghua (Hunan province) as a means for early detection of SRBSDV outbreaks. 

**Figure 10:**
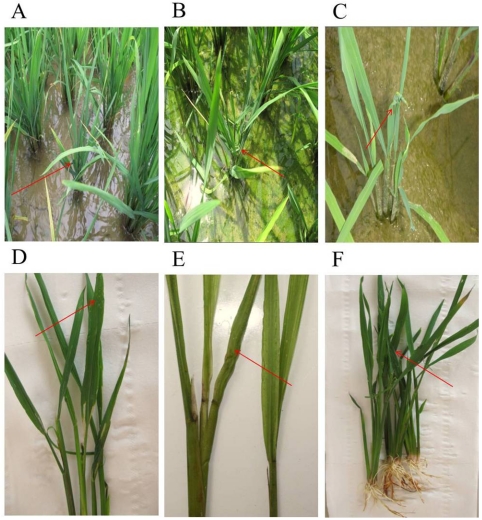
Typical characterization of tillering stage rice plants infected with SRBSDV from three county laboratories.

**Figure 11:**
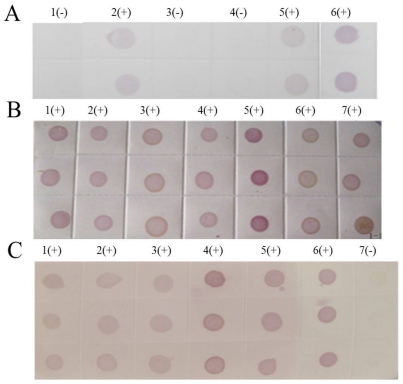
Results in three county laboratories. **(A)** Libo of Guizhou province. 1(-): Negative control (stem tissue). 2(+) - 5(+): Suspected samples. 6(+): Positive control. **(B)** Shidian of Yunnan province. 1(+) - 6(+): Suspected samples collected from Shidian in Yunan province. 7(+): Positive control. **(C)** Jianghua of Hunan province. 1(+) - 5(+): Suspected samples. 6(+): Positive control. 7(-): Negative control (stem tissue).

All samples picked from Libo, Shidian and Jianghua had the typical symptoms of SRBSDV disease, such as serious dwarf and wrinkled characteristic on leaves (Figure 10). The results from the three county laboratories showed evident purple-positive spots, indicating the presence of SRBSDV disease in Libo, Shidian and Jianghua (Figure 11). Furthermore, this fast check-test technology provided accurate and efficient data for local plant protection units to apply agriculture measures to control the spread of SRBSDV.

### 2.4. Discussion

At present, there is no effective and convenient method for SRBSDV detection in the field. Although PCR-based technologies reliably detect SRBSDV, these methods are time-consuming, laborious, and expensive, and impractical for local field detection. We developed an economical, quick and easy SRBSDV diagnosis kit. We obtained highly specific S10 polyclonal antibodies by the synthetic polypeptide technology, and measured them by Western blot experiments. Hence, a new rapid dot-ELISA method was improved with S10 polyclonal antibody 1. To further improve the reaction intensity from the background noise, different dilution ratios (1:1000 and 1:1500) in blocking buffer and incubation time were tested. Blocking the strips with 5% skim milk for 40 min at 37 °C produced the best results when SRBSDV antigen preparation was utilized. Therefore, for further testing, dot-ELISA was performed using SRBSDV antigen with 5% skim milk as the blocking agent to detect the SRBSDV antiserum. Of the different time periods tested for antiserum and conjugate (sheep anti-rabbit alkaline phosphatase), incubation of strips for 50 min in antiserum and 30 min in AP-conjugated secondary produced the best results. By means of these improvements, the assay for detection of SRBSDV infection could be finished within 3 hours.

In order to test the accuracy of dot-ELISA test, it was compared to the One Step RT-PCR method (Figure 3). The tested rice samples were picked from Shidian county of Yunnan province, and these rice plants exhibited typical symptoms of infection, including stunting and dwarfing with stiff and wrinkled leaves. For the rice from Shidian, Yuan Jiang still in the tillering stage, the virus content was much lower as evidenced by the lighter purple spots; nonetheless, they were easily distinguished from the negative controls. This dot-ELISA method could accurately distinguish positive from negative samples as well as the One Step RT-PCR assay. 

Both membranes (NC and PVDF) were equally suitable as revealed by direct comparison, but the NC membrane was less expensive, and so was used for most tests. The samples from Tianzhu in Guizhou Province and Liangping in Chongqing City were tested using both membranes in parallel experiments and both detected the presence of virus. In some cases, low levels of virus in seedling stage plants were likely to yield false-negatives, so it was necessary to use the higher specificity PVDF membrane. In some samples, the interference from chlorophyll, especially during the tillering stage of rice, interfered with the signal. In such cases, the blotted PVDF membranes could be washed in methanol with gently shaking for 15 to 30 seconds to remove most of the chlorophyll and other pigments to reduce the background interference.

This fast detection method was used in three county laboratories built in Libo (Guizhou province), Jianghua (Hunan province) and Shidian (Yunnan province), and these tests proved sufficiently accurate and sensitive for the early local detection of SRBSDV infections, allowing local officials to take appropriate responses to control spread. With the help of the early warning of SRBSDV infection, antiviral agent and insecticide were timely sprayed in local districts Libo, Jianghua and Shidian. The output results indicated decreased amounts of WBPHs and effectively controlled SRBSDV was ensured in these areas.

The causes and outbreak situation of SRBSDV infection were analyzed. Since 2010, serious SRBSDV outbreaks have occurred in Vietnam. The Chinese outbreaks may be due to WBPHs migrating from Vietnam, Myanmar, and Laos, all of which have had outbreaks. Guizhou, Sichuan, and especially Yunnan were hit by serious drought in 2011, which was particularly serious in central and eastern Yunan. Due to the drought and low winds, WBPH could not migrate, aggravating the SRBSDV outbreak in Yunnan in 2011. All bordering regions, such as Xuyong, Hejiang, Luodian, Congjiang, Guangnan, and Malipo, in addition to Chongqing city had outbreaks of SRBSDV dwarf disease (Figure 2). The huge natural barrier formed by the surrounding mountains may prevent the WBPHs from migrating further, leading to local insect accumulation and SRBSDV outbreaks.

We report a new simple dot-ELISA method for the rapid detection of SRBSDV. All incubation steps are performed at 37 °C in a water bath shaker with gentle shaking, and the results can be seen by the naked eye, so precise instruments and trained researchers are not required. The test is applicable to diagnose infection in the field as well as in laboratories which are not well equipped. Compared to the One Step RT-PCR assay, the dot-ELISA method greatly simplifies the procedure, shortens the detection time, and saves on materials. It is simple enough for high throughput analysis of hundreds of samples per day. To further improve the sensitivity and specificity of this assay, investigations on developing higher avidity and specificity monoclonal antibodies are currently under way.

## 3. Experimental Section

### 3.1. Suspected samples and Controls

Several suspected SRBSDV rice plants collected in June 2011 from Shidian, Yuanjiang and Malipo counties of Yunnan Province were used to compare results between the dot-ELISA method and the One Step RT-PCR assay. Suspected SRBSDV rice samples with typical dwarf symptoms were picked from Lincang, Chuxiong, Menghai, Kaiyuan, Jinghong, Shizong, Yongshan, Mile, Pingbian, Mengzi, Mengla, Shiping, Gejiu, Jianshui, Yiliang, Xinping, Fengqing, Ludian, Kunming, Shuifu, Zhenxiong, Yanjin, Maguan, and Guangnan in Yunnan province, Tianzhu, Luodian, Dushan, Huishui, and Kaiyang in Guizhou province, Liangping in Chongqing city and Jiajiang, Fushun and Hejiang in Sichuan province in June and July 2011. The plant tissues were frozen and stored at -20 °C. The SRBSDV test results from all samples are listed in Table 1. The samples tested in the three county laboratories were picked from local rice fields except the Shidian county laboratory also tested samples from Dehong county in Yunnan province. The positive control samples were stored rice plants from Libo county in Guizhou province confirmed to be infected with SRBSDV in 2010. The negative control sample was obtained from rice plants growing in a greenhouse.

**Table 1:** SRBSDV incidence of suspected samples from Southwestern China

Total	Districts (Province and City)						
	**Sichuan**	**Chongqing City**	**Guizhou**	**Yunnan**	**Guangxi**	**Jianghua**	**All specimens**
	**(n=8)**	**(n=1)**	**(n=9)**	**(n=38)**	**(n=4)**	**(n=1)**	**(n=61)**
	+ -	+ -	+ -	+ -	+ -	+ -	+ -
	7 1	1 0	7 2	35 3	4 0	1 0	55 6
Incidence	87.50℅	100℅	77.78℅	92.11℅	100℅	100℅	90.16℅

### 3.2. Dot-ELISA Methods

The solutions used were carbonate buffer (1000 mL distilled water, 1.59 g sodium carbonate, 2.93 g sodium bicarbonate, TBS (20 mM Tris-HCl, 0.15 M NaCl, pH 8.0), TBST (TBS with 0.05℅ Tween-20), blocking buffer (5℅ skimmed milk in TBST), antibody buffer (1:1500-fold dilution of antiserum in blocking buffer), and alkaline phosphatase buffer (0.1 M Tris-HCl, 0.1 M NaCl, 0.05 M MgCl2, pH 9.5). Color development solution for alkaline phosphatase containing 0.083 mg/mL nitro blue tetrazolium (NBT, Solarbio Technology, Beijing) and 0.05 mg/mL 5-bromo-4-chloro-3-indolyl phosphate p-toluidine salt (BCIP, Solarbio) was prepared just before use by mixing 50 mg NBT in 1 mL N, N-dimethylformamide for 1 to 2 min and mixing 50 mg BCIP in 1 mL N, N-dimethylformamide by vortexing for 5 min.

Virus antigen was purified from a clarified extract of rice infected with SRBSDV. The extract was made by grinding about 0.5~1.0 grams leaf, stem, and root tissue in 1.5~3.0 mL carbonate buffer. The debris was removed by centrifugation (10 min, 12000×g, 4 °C). The supernatant was removed and stored at -20 °C. 

The SRBSDV S10 polyclonal antibody was designed by our group [13] (Table 2). The polypeptide was synthesized in GLS (Shanghai, China) based on the S10 sequence, which was designed by using BLAST in NCBI Gen bank. The synthetic peptides corresponding to S10 were conjugated with keyhole limpet hemocyanin (KLH). New Zealand rabbits were immunized with 100 μg of the polypeptides. The polyclonal antibody was purified from the whole anti-SRBSDV rabbit antiserum and stored at -20 °C. Then the polyclonal antibody was diluted 1:1500-fold in blocking buffer when used in dot-ELISA assay.

**Table 2:** Information on the polypeptide sequences of the SRBSDV antigens

Protein	No.	Protein polypeptide fragment of SRBSDV	Synthetic polypeptide sequence
**S10**	1	20–39	CRNDQPTRNTNLSLSQSTENR
	2	140–159	CKDQKDDESQRPTSTDSTKNE
	3	319–339	CKRYRRFRTRIVGNADSVIKSD

The second antibody solution was a sheep anti-rabbit IgG conjugated to alkaline phosphatase (Wuhan Boster Biological Technology Co., Ltd, China) diluted 1:30000-fold in blocking buffer used in dot-ELISA method. In western blot assay, the secondary antibody was horseradish peroxidase-conjugated.

### 3.3. Evaluation of polyclonal antibodies

The artificially synthesized polypeptide antigen was incubated overnight at 4 °C. Bovine serum albumin (BSA; 2%) in phosphate-buffered saline with Tween20 (PBST) buffer was added. The system was closed at 200 μL/well and 37 °C for 1-2 h. The diluted antisera were hatched at 37 °C for 1–2 h. Then a second incubation with alkaline phosphatase (AP)-conjugate secondary antibody was performed at 37 °C for 1–2 h. The samples were washed after each step with PBST. The optical density (OD), stained by a 3, 3', 5, 5'-tetramethylbenzydene system, was measured using a microplate reader at 450 nm. The diluted titers of the antisera were then analyzed.

The western blot assay procedure for determination of three S10 polylnal antibodies was as follows. The obtained SRBSDV protein supernatant was collected and separated by 12% SDS-PAGE electrophoresis. Separated proteins in the gels were elctrophoretically onto the PVDF membrane at 90 mA for 1.5 h. The blotted membrane was blocked with 5% skim milk in TBST buffer. After washing the membrane three times with TBST for 15 min, the membrane was incubated with polyclonal antibodies at 5 μg·mL^-1^. The bound antibodies were detected by horseradish peroxidase 1:3000-fold dilution.

### 3.4. Dot-ELISA testing procedure

For the following procedures, all steps were performed at 37 °C in a shaker water bath. Individual dilutions of the antigen were applied directly onto PVDF membrane (0.22 μm, Millipore Company, U.S.) or NC membrane (0.22 μm, PALL Company, U.S.) in different lines. Sheets were always handled by forceps and vinyl gloves. If necessary, the PVDF membrane with sample dots was soaked in methanol with gently shaking for 15 to 30 s to remove most of the chlorophyll and other pigments to reduce the background interference. Membranes were blocked for 40 minutes with 5% skim milk solution in blocking solution (TBST). Serum samples were applied at 1:1500 dilutions and incubated together with the test strips for 50 min. Following three washing steps of 3 min each, a second incubation of 30 min with alkaline phosphatase (AP)-conjugate secondary antibody (sheep anti-rabbit IgG) was performed. After three additional washing steps of 3 min each, strips were stained for about 10 min using NBT and BCIP, and the reaction stopped by washing blots in distilled water. Controls blots were used as described previously. The appearance of purplish-red dots was observed with the naked eye. The wet sheets were observed in nature light and a well-defined purple-red spot was regarded as positive. The sheets could be stored dry and re-wetted in distilled water to restore the color intensity.

### 3.5. One Step RT-PCR Methods

The total RNA of suspected SRBSDV rice plants was extracted according to the RNAiso plus total RNA manual (TAKARA, Japan). The OD_260 _and OD_280_ of the RNA extraction were determined on an ultraviolet spectrophotometer, and the total RNA purity value was estimated by the OD_260_/OD_280_. All samples used had OD_260_/OD_280 _values between 1.8 and 2.2. The concentration of total RNA was calculated according to the dilution ratio and the value of OD_260_, and diluted to 1 μg/mL with DEPC water.

According to the SRBSDV dsRNA segment 10, a pair of primers, S10F/S10R, was designed and synthesized by TAKARA (Table 3).

**Table 3:** Two pairs of unique primers of SRBSDV S10

Number	left primer	right primer
1	5’- ctc,cgc,tga,cgg,ttt,aga,ag -3’	5’- ggt,cgt,aac,cgc,cat,agt,gt -3’

The One Step RT-PCR (PrimeScript® One Step RT-PCR Kit Ver.2, TAKARA, Japan) reaction solution contained 1 μL Primer Script Enzyme Mix, 12.5 μL 2×One Step Buffer, 1 μL S10-R, 1 μL S10-F, 1 μL extraction RNA as template, and 8.5 μL RNase free water. Reverse transcription was performed at 50 °C for 30 min, followed by 30 cycles of 94 °C for 2 min, 94 °C for 30 s, and 58 °C for 30 s, followed by a final extension at 72 °C for 50 s. The One Step RT-PCR products were analyzed by 2.5% agarose and 8℅ PAGE gels.

## 4. Conclusions

Although the southern rice black-streaked dwarf virus (SRBSDV) was identified in 2008, detection of infection has depended on RT-PCR methods. Economic losses from SRBSDV outbreak were so serious that the development of a new rapid SRBSDV diagnostic method to detect the virus at the early stages of infection was urgently needed to allow sufficient time for preventative measures to avoid the spread of the virus. We found that three provinces and one city in southwest China had SRBSDV outbreaks in 2011, underscoring the urgent need for a rapid test that can be used under field conditions.

The dot-ELISA assay may be more suitable for routine detection of SRBSDV because it is fast, inexpensive and user-friendly. This is the first report that has used a dot-ELISA to diagnose SRBSDV infection. With this method, SRBSDV could be detected in several milligrams of infected rice tissue within 3 hours. Based on our detection data, we found that 55 districts out 61 sampled had an outbreak of SRBSDV disease, indicating the seriousness of the blight to China.

This simple, rapid and sensitive assay was proven to be a useful and practical diagnostic technique for plant viral diseases and could be applied for detecting suspected cases in county laboratories. According to the results of the three established county laboratories, this dot-ELISA assay provided a new rapid detection system. If there were much more county laboratories established in China, agricultural technicians or even farmers themselves could test their own crops for dwarf rice disease. Early detection of SRBSDV disease could greatly aid measures aimed at preventing disease spread.
